# Shared experiences and prosocial responding: the roles of self-other overlap and perceived emotional synchrony

**DOI:** 10.3389/fpsyg.2026.1763805

**Published:** 2026-10-07

**Authors:** Xiaodi Xue, Junkai Ye, Yueying Lee, Song Tong, Kaiping Peng

**Affiliations:** 1 Department of Cognitive and Psychological Sciences, Tsinghua University, Beijing, China; 2 Department of Psychology, School of Public Policy and Management, Nanchang University, Nanchang, China; 3 Faculty of Business and Management Beijing Normal-Hong Kong Baptist University Zhuhai, Zhuhai, China; 4 Bay Area School of Applied Psychological Sciences, Beijing Normal University at Zhuhai, Zhuhai, China; _5_ Beijing Key Laboratory of Applied Experimental Psychology, National Demonstration Center for Experimental Psychology Education (Beijing Normal University), Faculty of Psychology, Beijing Normal University, Beijing, China

**Keywords:** collective experience, perceived emotional synchrony, prosocial responding, self-other overlap, shared experience

## Abstract

Social isolation and difficulties in self-related processing have been linked to a range of psychological challenges. While shared experiences (from collective grief to celebratory events) are known to strengthen social bonds, the mechanisms through which they may beneficially influence self-related cognition and promote prosocial responding remain underexplored. This study investigates perceived emotional synchrony (PES)—the subjective sense of emotional convergence with others during collective events—as a key socioemotional process linking shared experiences to prosocial responding and shaped by self-other overlap (SOO), a core indicator of relational-cognitive connection between the self and others. Across four progressive studies involving both positive and negative emotional contexts, we investigated how shared experiences related to changes in self-perception and subsequent prosocial responding (as an indicator of social function). The results indicated that: (1) sharing an emotional event was associated with greater prosocial responding than individual experience across both positive and negative contexts; (2) in negative emotional contexts, SOO may function as an upstream relational-cognitive state associated with higher levels of perceived emotional synchrony; (3) this cognitive-affective pathway partially accounted for the prosocial effects of shared negative experience. Additional analyses were more consistent with the proposed serial model in which SOO preceded PES than with the reversed serial model. Together, these findings provide a refined account of how shared experiences may promote prosocial responding through cognitive-affective processes that shape the relationship between the self and others.

## Introduction

Recent research suggests that shared experiences can promote intragroup cooperation and social bonding across contexts involving synchronous action, physiological pain, and social rejection (Wiltermuth and Heath, 2009; Bastian et al., 2014; Miao et al., 2020). But shared experiences in everyday life are different from these relatively structured forms of co-experience. They are emotional in nature yet occur under relatively incidental and low-interaction conditions (Shteynberg et al., 2014), such as watching a film, listening to music (Zentner et al., 2008), attending a public ritual (Zou et al., 2018), or living through a collective event alongside others (Páez et al., 2015). In such situations, individuals may not engage in sustained interaction, explicit coordination, or prior social exchange, yet the experience may still influence how they perceive and respond to others (Echterhoff et al., 2009). Clarifying this process may help explain how transient shared experiences reduce momentary social distance and support adaptive social functioning.

Prior studies have largely documented the social outcomes of shared experience; however, the underlying psychological processes remain insufficiently understood. One possibility is that shared experiences influence how individuals represent themselves in relation to others (Aron et al., 1992). In this regard, Self–Other Overlap (SOO) may serve as a key relational-cognitive process. SOO refers to a perceived reduction in the boundary between the self and others, reflecting a shift from a more isolated to a more relational mode of self-representation (Aron et al., 1992; Wlodarczyk et al., 2020). It is commonly assessed using the Inclusion of Other in the Self (IOS) Scale, which captures the perceived overlap between self and other representations. In the context of shared experiences, such overlap may arise when individuals perceive others as being “in the same situation as me”. This shift may increase the psychological relevance of others and create conditions under which social responses become more likely.

At the same time, individuals in shared experiences often report a sense of emotional convergence with others (Pizarro et al., 2022), referred to as perceived emotional synchrony (PES). PES captures the subjective experience of sharing emotional states with others during collective events (Páez et al., 2015). We propose that SOO and PES may not merely be parallel consequences of shared experience but may also occupy different positions in a cognitive-affective sequence. SOO reflects a relational representation of how the self is positioned relative to others. When self-other boundaries become less distinct, others may become more psychologically relevant, making their internal states more salient and increasing individuals’ attention and receptivity to their emotional cues. This heightened interpersonal relevance may, in turn, facilitate the incorporation of others’ emotional states into one’s own emotional processing, thereby increasing the likelihood of perceived emotional convergence.

Importantly, we do not assume that SOO necessarily produces PES, nor that the two processes cannot co-occur. Rather, greater SOO may provide an upstream relational-cognitive condition that facilitates the emergence of PES. This account is consistent with theoretical accounts emphasizing the role of self-other representation in emotional alignment (Hatfield et al., 1993; Niedenthal, 2007). Recent work suggests that self-representation can shape emotional contagion, while affect-sharing is influenced by top-down cognitive, situational, and relational factors (Wang et al., 2024; Gamble et al., 2024). Related evidence has also shown a serial mediation pattern in which self–other overlap was positioned upstream of interpersonal neural synchronization in the association between behavioral synchrony and prosociality (Feng et al., 2020). Accordingly, SOO may function as a more upstream relational-cognitive condition, whereas PES may represent a more proximal socioemotional process associated with prosocial responding.

Based on this cognitive-affective framework, the current study investigates how collective shared experiences relate to self-related cognition and social functioning. Rather than examining isolated associations, we test the cognitive-affective pathway linking shared experience to prosocial responding. Specifically, this study addresses three related questions. First, do shared emotional events—both positive and negative—promote prosocial responding among co-experiencers? Second, are shared experiences associated with increased SOO, reflecting a shift in relational-cognitive representation? Third, are the prosocial consequences of shared experience consistent with a cognitive-affective pathway in which greater SOO is associated with higher PES, which in turn relates to greater prosocial responding?

## Study 1a: the effect of shared positive experience on prosocial responding

The aim of this study is to examine the effect of experience mode (shared vs. individual) on prosocial responding toward experimental partners under positive emotional valence. This experiment employed a laboratory design in which experience mode (shared vs. individual) was manipulated, while positive emotional valence was controlled through musical stimuli. Based on social cohesion theory and the broaden-and-build theory of emotions, we anticipate that the shared experience will lead to higher cooperation.

### Participants

A between-subjects design was employed with experience mode (shared vs. individual) as the independent variable. Assuming an effect size of 0.5, an alpha level of 0.05, and a desired power of 0.80, the G*Power analysis indicated that a minimum of 120 participants was required. The final sample consisted of 129 university students (25 male, 104 female; *M* = 20.53, *SD* = 1.82).

### Materials

The experimental materials included an instrumental excerpt selected from Handel’s Water Music to induce a positive emotional state, a state emotion self-assessment scale, and behavioral measurement tools. State Emotion Self-Assessment was measured using a single 7-point scale (0 = not at all, 6 = extremely) to assess “To what extent do you currently feel joyful?” (Zentner et al., 2008).

Interpersonal perception was assessed by a 3-item scale adapted from Wiltermuth and Heath (2009). This instrument assessed participants’ subjective perceptions of similarity, connectedness, and trust toward their group members.

The primary dependent variable, Prosocial Behavior, was measured via the Weak-link Coordination Game. In this game, each participant’s payoff depends on the intersection of their own choice and their partner’s choice (see Table 1). Choosing a higher number indicates a higher level of cooperation toward others and can lead to a higher payoff. Consistent with previous research, cooperative behavior was measured by the initial choice (round 1 value) and total earnings across all six rounds (Weber et al., 2004; Weber et al., 2001; Wiltermuth and Heath, 2009). Additionally, participants’ familiarity with and preference for the music were measured as control variables.

**Table 1:** Payoff matrix for the weak-link coordination game.

The number participant choose	The minimum number among group members						
	7	6	5	4	3	2	1
7	13 RMB	11 RMB	9 RMB	7 RMB	5 RMB	3 RMB	1 RMB
6		12 RMB	10 RMB	8 RMB	6 RMB	4 RMB	2 RMB
5			11 RMB	9 RMB	7 RMB	5 RMB	3 RMB
4				10 RMB	8 RMB	6 RMB	4 RMB
3					9 RMB	7 RMB	5 RMB
2						8 RMB	6 RMB
1							7 RMB

Payoffs increase as a function of the smallest number chosen by a group member and decrease with the distance between the participant’s choice and the minimum value chosen in the group, RMB refers to Chinese yuan.

### Procedure

Participants were randomly assigned to groups (shared or individual experience) of three and informed that they would take part in a music evaluation experiment, during which they could freely express their emotions. In the shared experience condition, the three participants were explicitly told that they would listen to the exact same piece of music together, with the experimenter broadcasting the music using a computer and speakers. In the individual experience condition, each participant was guided to a separate cubicle within the same lab where they listened to the music via headphones on individual computers. Although the musical stimulus was in fact identical across participants, they were not told this; they only knew that the other participants were also listening to music. Participants in both groups were informed that they could express their emotions spontaneously during the listening session, but they were not permitted to communicate or interact with one another.

After listening, participants first reported emotions, followed by the Weak-link Coordination Game. Subsequently, they completed the remaining questionnaires before being debriefed and compensated. No missing data were observed for the key study variables.

## Results

Independent samples t-tests indicated no significant differences between conditions regarding induced pleasure, music familiarity, music preference, trust or connectedness (all *p*s > 0.33).

However, a significant difference emerged in perceived similarity, *t*(127) = 3.21, *p* < 0.01, Cohen’s *d* = 0.57. Specifically, participants in the shared experience group perceived greater similarity with their team members compared to those in the individual group. Regarding the Weak-link Game, significant differences were observed in both the first-round numerical choice and total earnings (see Table 2).

**Table 2:** Effects of experience mode on prosocial behavior (weak-link game).

Outcome	Experience mode	*M*	*SD*	*t*	Cohen’s *d*
First-round choice	Shared experience	5.17	1.73	2.505^*^	0.44
	Individual experience	4.42	1.67		
Total earnings	Shared experience	51.63	12.65	2.284^*^	0.41
	Individual experience	46.65	12.13		

^*^p < 0.05, ^**^p < 0.01. Experience Mode: 0 = Individual Experience, 1 = Shared Experience.

## Discussion

These results from Study 1a indicate that exposure to shared positive emotional experiences was significantly associated with greater perceived similarity and prosocial cooperation compared to individual positive experiences. These findings align with the “social glue” hypothesis (Wheatley et al., 2012) and are consistent with prior research documenting the positive effects of co-experience on interpersonal similarity (Rabinowitch and Knafo-Noam, 2015) as well as group cooperation (Miao et al., 2019; Reddish et al., 2013).

Notably, a nuanced pattern emerged in which shared experience condition was associated with greater perceived similarity—a cognitive construct—while showing no significant effects on trust or connectedness. This pattern may reflect that shared experiences have a stronger impact on cognitive aspects of social perception, such as perceived similarity, which has been linked to processes of shared reality (Echterhoff et al., 2009). In contrast, more affective indicators of social bonding, such as trust and connectedness, may be less readily influenced in this context and may depend on additional relational or interactional cues (Higgins, 2019).

In addition, it is possible that the post-game assessment context contributed to variability in affective responses. Emotional reactions such as trust and connectedness may be more sensitive to immediate outcomes and feedback within the task (Camerer, 2003), whereas perceived similarity may represent a relatively more stable cognitive evaluation (Montoya and Horton, 2004).

## Study 1b: Shared negative experience and prosocial responding: evidence from a real-world event

Study 1b extends the investigation by examining whether the association between shared experience and prosocial responding extends to naturalistic contexts involving negative emotional events. In contrast to the controlled laboratory setting of Study 1a, the present study focuses on real-world shared experiences. We hypothesize that shared negative emotional experiences will likewise be associated with greater prosocial responding than individual experiences.

### Participants

A total of 167 responses were initially collected 6 days after Qingming Festival in 2020. After excluding 2 responses due to failed attention checks, the final sample consisted of 165 participants. Participants were recruited online through Wenjuanxing and completed the survey for a small monetary compensation. Participants ranged in age from 18 to 50 years (*M* = 22.45, *SD* = 4.71). The sample consisted of 61 males and 104 females.

### Materials

#### Objective participation and experience mode

The experience mode (shared vs. individual) was determined by objective participation in the national siren mourning event held during the Qingming Festival, which commemorated individuals who lost their lives during the COVID-19 pandemic.

The Objective Participation Questionnaire assessed whether participants engaged in the collective mourning behavior at 10:00 a.m. on Qingming Festival. This was operationalized by two binary (“yes”/"no”) items: “At 10:00 AM on Qingming Festival, did you participate in activities such as observing silence, siren-blowing, or ceremonial viewing?” and “On Qingming Festival, did you witness or hear collective mourning behaviors and sounds?” Each “yes” was scored 1 point. Participants with a final score of 2 points were coded as the shared experience group, while those with score below 2 points were coded as the individual experience group. The Mourning Video, a 3-min video depicting nation-wide moments of mourning at 10:00 a.m. on Qingming Festival, was utilized as a stimulus to facilitate emotional recall and contextualization, ensuring participants were focused on the shared event.

#### Emotional and social measures

Retrospective emotion during the Qingming Festival was assessed using the Chinese version of the Positive and Negative Affect Schedule (PANAS; Qiu et al., 2008; Watson et al., 1988). The Social Desirability Scale (Reynolds, 1982) was used to assess participants’ tendency to overreport prosocial behavior.

#### Prosocial behavior measurement

Prosocial behavior was assessed retrospectively using a self-report checklist of common COVID-19–related prosocial behaviors. Participants indicated which actions they engaged in following the Qingming Festival national mourning event.

## Results

No missing data were observed for the key study variables. Correlation analysis of the 165 valid responses revealed that prosocial behavior was significantly correlated with age, the experience mode, and social desirability (see Table S5 in Supplementary Materials). Specifically, the experience mode (shared vs. individual) was positively correlated with actual prosocial acts and self–other overlap (SOO).

To further examine the effect of experience mode on prosocial behavior and SOO, hierarchical regressions were conducted, age and social desirability were entered in the first step, and experience mode was entered in the second. After controlling for covariates, experience mode remained a significant positive predictor of actual prosocial behavior and SOO (see Table 3). These results suggest that participation in the shared mourning ritual was associated with higher levels of prosocial behavior in the days following the event.

**Table 3:** Hierarchical regression analysis predicting prosocial behavior and self-other overlap (SOO).

Predictors	Prosocial behavior			SOO		
	*B*	95% CI [LL, UL]	*β*	*B*	95% CI [LL, UL]	*β*
Step 1: Covariates						
Age	0.04	[−0.001, 0.088]	0.15†	0.00	[−0.047, 0.059]	0.02
Social desirability	0.08	[−0.013, 0.183]	0.13†	0.00	[−0.113, 0.121]	0.01
Step 2: Main Effect						
Age	0.04	[−0.005, 0.082]	0.13†	0.00	[−0.051, 0.054]	0.01
Social desirability	0.06	[−0.042, 0.155]	0.09	−0.02	[−0.139, 0.098]	−0.03
Experience mode	0.60	[0.174, 1.022]	0.25^**^	0.51	[0.001, 1.023]	0.16^∗^
*R* ^2^	0.09			0.02		
Δ*R*^2^	0.044^**^			0.024^∗^		

^†^*p* < 0.10, ^*^p < 0.05, ^**^p < 0.01; Experience Mode: 0 = Individual Experience, 1 = Shared Experience.

## Discussion

The findings of Study 1b provide support for Hypothesis 1b. The shared negative experience was significantly associated with higher frequencies of prosocial acts and greater SOO compared to the individual experience. The results suggest that the prosocial effect of shared experience may extend to naturalistic negative emotional contexts.

However, several limitations should be noted. First, the retrospective design may introduce memory bias and *post hoc* reconstruction, potentially affecting reports of emotional intensity and prosocial acts. Second, although SOO was linked to shared experience, the broader psychological mechanisms underlying this association were not tested.

To address these limitations, the subsequent study moves beyond retrospective reports by examining shared experience during an ongoing collective event. It further examines whether SOO is associated with PES and whether PES is linked to prosocial responses in an ongoing collective crisis.

## Study 2: investigating the predictive role of perceived emotional synchrony (PES) in real-world crisis

Although prior research has identified PES as an important socioemotional process in collective experience, it remains unclear how PES operates among loosely connected individuals in ongoing shared adversity. In particular, it is not clear whether emotional synchrony emerges directly from shared experience, or whether it depends on prior relational-cognitive alignment between self and others.

Study 1b provided preliminary evidence that shared negative experience was associated with greater SOO. Building on this finding, the present study investigates whether SOO is related to PES in a real-world crisis context, and whether PES is associated with prosocial responses.

We hypothesize that higher levels of PES will be associated with greater prosocial responding, and that SOO will be positively related to PES.

### Participants

This study adopted a survey design, with data collected via the Credamo platform. Data collection was conducted during the Zhengzhou rainstorm, specifically on the fifth day after its onset, as earlier data collection was not feasible due to severe disruptions in communication networks caused by the disaster. All participants were located in Zhengzhou at the time of survey administration, as confirmed by mobile IP tracking. The rainstorm was still ongoing and public transportation remained suspended.

A total of 380 responses were collected, of which 362 passed the attention check. The final sample of 362 participants included 211 males and 151 females, with ages ranging from 18 to 59 years (*M* = 28.57, *SD* = 6.35).

### Materials

#### Experience and emotion measures

The study utilized the Objective Experience Survey for screening, aiming to confirm whether participants experienced the Zhengzhou rainstorm, thus establishing the shared negative event context. PES, a key socioemotional variable in the present study, was assessed using a revised 15-item scale developed by Páez et al. (2015), which demonstrated high internal consistency in the present study (α = 0.91). The Positive and Negative Affect Schedule (PANAS) was employed to measure the emotional intensity during the Zhengzhou rainstorm.

#### Social-cognitive and covariate measures

The key cognitive variable, SOO, was measured to assess the participants’ perceived overlap between self and others following the shared event. Additionally, given the context of a natural disaster, several potential confounds were measured as covariates. State Empathy was assessed using a 6-item questionnaire (Feng et al., 2020), which included items like “feeling compassionate” and “wanting to comfort them,” due to its known influence on prosociality. Participants’ current Need for Connection was also measured using an adapted 3-item version of the State Need to Belong scale (Lv et al., 2021; Zawadzki, 2009), assessing their desire to establish and feel closer to others who experienced the rainstorm.

#### Prosocial behavior measures

Prosocial behavior was measured across two domains: Actual Prosocial Behavior and Prosocial Intention. Actual prosocial behavior was retrospectively delineated into specific categories of support—instrumental support (e.g., donating supplies), emotional support (e.g., comforting victims), and informational support (e.g., forwarding rescue information). Prosocial Intention was measured via specific questions contextualized within real-world scenarios, adapted from established paradigms (Garcia et al., 2002). Participants indicated their willingness to dedicate free time to voluntary aid (e.g., clearing street debris) and their willingness to donate a percentage of their survey compensation or hypothetical annual income to the rainstorm-affected areas in Zhengzhou, using a sliding scale from 0 to 100%.

#### Procedure

Because the Zhengzhou rainstorm resulted in severe casualties and widespread disruption of infrastructure, data collection could not be conducted immediately following the onset of the disaster due to interruptions in communication networks. Data collection therefore began on the fifth day after the rainstorm first struck and was completed within 1 week. Importantly, data were collected while the rainstorm was still ongoing and rescue efforts were actively underway, allowing participants to report their experiences within an ongoing shared adversity context rather than relying on distant retrospective recall.

To ensure data validity and context relevance, the terminal device IPs were strictly restricted to Zhengzhou City. The survey sequence began with the Objective Experience Survey, followed by the main measures (PES, SOO, PANAS), and concluded with the prosocial behavior and intention measures, and covariate scales. No missing data were observed for the key study variables.

## Results

### Correlations among covariates and outcome variables

A Pearson correlation analysis was conducted on the data from the 362 participants to investigate the associations between the control variables and the primary outcome metrics. The results indicated that demographic factors (age and gender) and psychological traits (state empathy and need for connection) were significantly correlated with the various prosocial behavior metrics. Consequently, these variables were retained as covariates and statistically controlled for in all subsequent predictive models to estimate the unique association of PES with prosocial responding.

### Predictive effects of pes on prosocial responses

To test the main predictive relationship, two-step hierarchical regression analyses were performed. Age, gender, empathy, and the need for connection were entered in Step 1, and PES was added in Step 2. The results revealed a mixed pattern across the subtypes of prosocial behavior. PES was not found to be a significant predictor for instrumental prosocial behavior (*B* = −0.01, *p* = 0.81, 95% CI = [−0.045, 0.035], Δ*F* = 0.06); or informational prosocial behavior (*B* = 0.01, *p* = 0.54, 95% CI = [−0.029, 0.055], Δ*F* = 0.38). But PES was a significant predictor for emotional prosocial behavior (*B* = 0.33, *p* < 0.01, 95% CI = [0.108, 0.543], Δ*F* = 8.691, Δ*R*^2^ = 0.018) and prosocial intention regarding volunteer time (*B* = 0.26, *p* < 0.05, 95% CI = [0.076, 0.447], Δ*F* = 7.663, Δ*R*^2^ = 0.019).

### Mediation analysis

A mediation analysis was conducted using PROCESS Model 4 with 5,000 bootstrap samples while controlling for age, gender, empathy, and need for connection. As shown in Figure 1, SOO significantly predicted PES, which in turn significantly predicted prosocial intention. The direct effect of SOO on prosocial intention remained significant after the mediator was included, indicating partial mediation. In addition, the total effect of SOO on prosocial intention was significant, and the indirect effect through PES was also significant (see Table 4).

**Figure 1:**
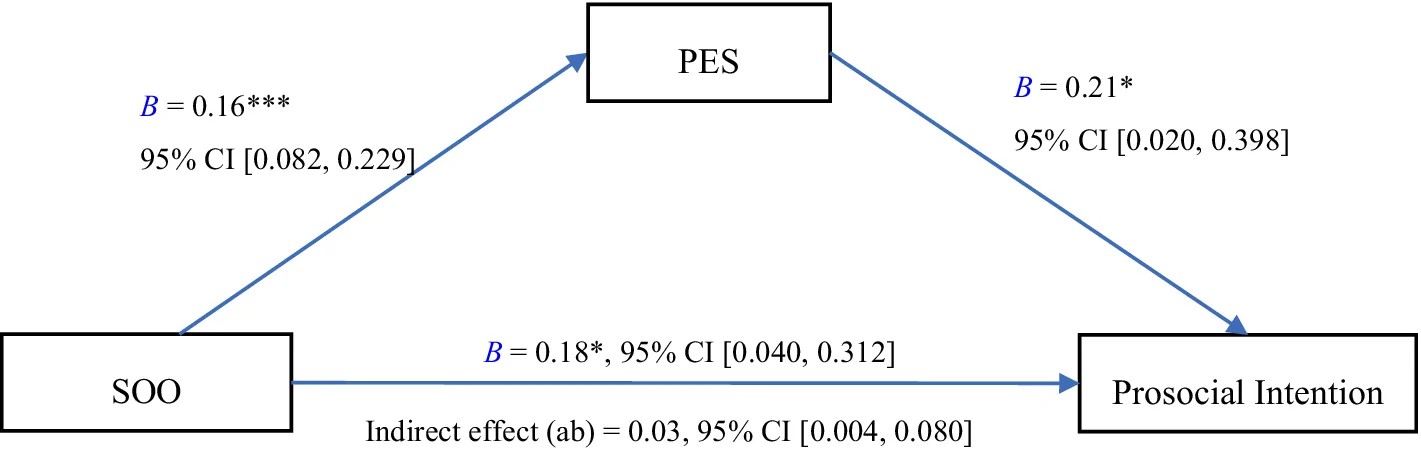
The mediating role of PES in the relationship between SOO and prosocial intention. Unstandardized coefficients are displayed.

**Table 4:** Summary of mediation analysis for the effect of soo on prosocial intention via perceived emotional synchrony (PES).

Path	Effect	SE	*t*	95% CI
SOO → PES	0.156	0.04	4.17	[0.082, 0.229]
PES → Prosocial Intention	0.209	0.10	2.18	[0.020, 0.398]
SOO → Prosocial Intention	0.176	0.07	2.54	[0.040, 0.312]
Total effect	0.208	0.07	3.07	[0.075, 0.342]
Indirect effect	0.033	0.02	—	[0.004, 0.080]

The indirect effects were tested on 5,000 bootstrap samples. Confidence intervals for the indirect effect are bootstrap confidence intervals.

## Discussion

Study 2 extended the previous findings by showing that, within a real-world context of shared adversity, PES was positively associated with specific forms of prosocial responding even after controlling for relevant demographic and psychological covariates. Although the effect size was modest, this pattern is theoretically meaningful. In real-world disaster settings, prosocial responses are shaped by multiple factors simultaneously, including practical constraints, perceived efficacy, empathy, and social motives (Staub and Vollhardt, 2008). Thus, the incremental association of PES with prosocial responding suggests that emotional synchrony may represent a meaningful proximal socioemotional process, even if its association is not uniform across different forms of prosocial behavior. This finding is consistent with prior research showing that shared emotional experiences and synchrony can enhance social bonding and cooperative tendencies (Wiltermuth and Heath, 2009; Páez et al., 2015).

The mediation analysis results further inform this interpretation. SOO was positively associated with PES, and PES, in turn, was associated with prosocial intention, yielding a significant indirect effect. This pattern is consistent with the proposed cognitive-affective framework, in which SOO may function as an upstream relational-cognitive condition associated with greater PES. Recent work suggests that self-representation shapes how others’ emotions are perceived and shared, and that affect-sharing is modulated by top-down cognitive and relational factors (Wang et al., 2024; Gamble et al., 2024).

At the same time, the direct effect of SOO remained significant, yielding a pattern consistent with partial mediation. This suggests that SOO may be associated with prosocial responding through multiple pathways in this context. Beyond facilitating emotional synchrony, SOO may also relate to greater perceived common fate or interpersonal relevance toward others undergoing the same event. This interpretation aligns with prior research suggesting that SOO can motivate prosocial behavior through identification and perceived shared identity (Aron et al., 1992; Cialdini et al., 1997; Zabala et al., 2024). Accordingly, PES may represent an important, but not exclusive, socioemotional pathway associated with the relationship between SOO and prosocial responding in this context.

Importantly, the different prosocial outcomes examined in Study 2 should not be regarded as psychologically interchangeable. The pattern of stronger and weaker associations should also be interpreted in light of both context and measurement. Different forms of prosocial responding may involve distinct motivational demands, behavioral costs, and situational opportunities, particularly in an ongoing crisis; accordingly, the strength of the association between PES and prosocial responding may vary across behavioral domains. In addition, SOO was measured using a single-item pictorial indicator, whereas PES was assessed with a multi-item scale, which may have contributed to PES emerging as a more sensitive proximal predictor in this study.

Nevertheless, because Study 2 relied on a correlational survey design conducted in a naturally shared-event context, causal inferences cannot be drawn regarding the relationships among SOO, PES, and prosocial intention. Although the results are consistent with the proposed cognitive-affective pathway, they do not establish the causal direction or temporal ordering of these associations. Thus, the contribution of Study 2 lies primarily in identifying PES as a meaningful socioemotional process associated with particular forms of prosocial responding within shared adversity and in providing ecological evidence for the association between SOO and PES. Building on this ecological evidence, Study 3 employs a controlled experimental design to more directly test whether shared experience causally influences SOO, PES, and prosocial behavior.

## Study 3: The chain mediation model via self-other overlap (SOO) and PES

Study 3 adopts a controlled laboratory experiment in which shared experience is directly manipulated. We hypothesize that participants in the shared experience condition will exhibit higher levels of prosocial responding than those in the individual experience condition. Furthermore, we hypothesize that this effect will be serially mediated by SOO and PES.

### Participants

Based on a G*Power analysis (*f* = 0.25, *α* = 0.05, Power = 0.95), a minimum of 210 participants (105 per condition) was targeted to replicate the effects observed in previous experiments. The final sample consisted of 209 participants (*M* = 22.71, *SD* = 2.83) after excluding incomplete responses, including 76 males and 133 females.

### Materials

#### Stimuli and covariates

The emotional stimuli included sadness-inducing video segments selected via a pilot study from films such as *Departures*, *Champion*, *and Go Away Mr. Tumor.* The duration of the videos ranged from 163 to 269 s. The neutral stimuli consisted of a 285 s video demonstrating ruler-and-compass construction. Pilot testing ensured the sadness segments possessed high levels of intensity and arousal (see Supplementary Materials). Participants rated their familiarity with and preference for the segments on a 5-point Likert scale, which were used as covariates.

#### Mechanistic measures

Positive and Negative Affect Schedule (PANAS), SOO, and PES were measured as in Study 2.

#### Prosocial behavior measure

Prosocial Behavior was quantified via the Public Goods Game (PGG) paradigm (Marwell and Ames, 1979), a standard measure of cooperative behavior. Participants were allocated 10 tokens and could choose how many to contribute to a public account versus a private account. If one token is invested in a public account, all participants (including oneself) will receive 1 RMB. If one token is invested in a personal account, only oneself will receive 2 RMB. The total number of tokens invested in the public account served as the metric for prosocial cooperation.

#### Procedure

Participants arrived at the laboratory in groups of eight (octads) and, after signing informed consent forms and being briefed on the procedure, were randomly assigned seat numbers to ensure randomization. To stabilize affect and establish baseline emotional levels, the entire group collectively watched the neutral video segment. Participants were then randomly assigned to either the shared experience or individual experience group for the main task. In the shared experience condition, the sadness-inducing stimuli were collectively viewed, with the video projected onto a screen; in the individual experience condition, participants viewed the same stimuli in separate cubicles with personal screens in the same room. All the participants were explicitly encouraged to express their emotions freely during the stimuli presentation, but they were not permitted to communicate or interact with one another. Following the viewing, participants immediately completed the questionnaires and then participated in the Public Goods Game (PGG), which served as the final measure of prosocial behavior.

## Results

### Missing data were minimal

Only the variable familiarity contained missing values (4 cases, 1.9%). Analyses involving familiarity were conducted using available data.

#### Manipulation checks

A paired-samples t-test was conducted to verify the emotional impact of the video stimuli, t(208) = 28.12, *p* < 0.001, Cohen’s d = 1.95, indicating that the video successfully induced negative emotional responses.

Independent samples *t*-tests were first conducted to ensure the experimental conditions were successfully implemented. The results confirmed that there were no significant differences between the shared and individual experience groups regarding either baseline affect or the level of video-induced sadness (*p* > 0.05 in both cases). Additionally, differences in participants’ preference for and familiarity with the video stimuli were not statistically significant (*p* > 0.05), suggesting that there were no significant differences in these variables.

#### Correlation analysis

Correlation analyses showed that shared experience, SOO, PES, and prosocial behavior were significantly correlated (see Table S10 in Supplementary Materials).

#### Experience mode on prosocial behavior

A significant difference between the two experience conditions was observed in PGG contributions, *t*(207) = 3.60, *p* < 0.01, Cohen’s *d* = 0.50.

#### Serial mediation analysis

Serial mediation analysis was conducted using PROCESS (Model 6, 5,000 bootstrap samples). As illustrated in Figure 2, the effect of shared experience on prosocial behavior was partially transmitted through a serial pathway from SOO to PES. Specifically, the serial indirect effect via SOO and PES was significant, whereas the indirect effects through SOO alone and PES alone were not (see Table 5).

**Figure 2:**
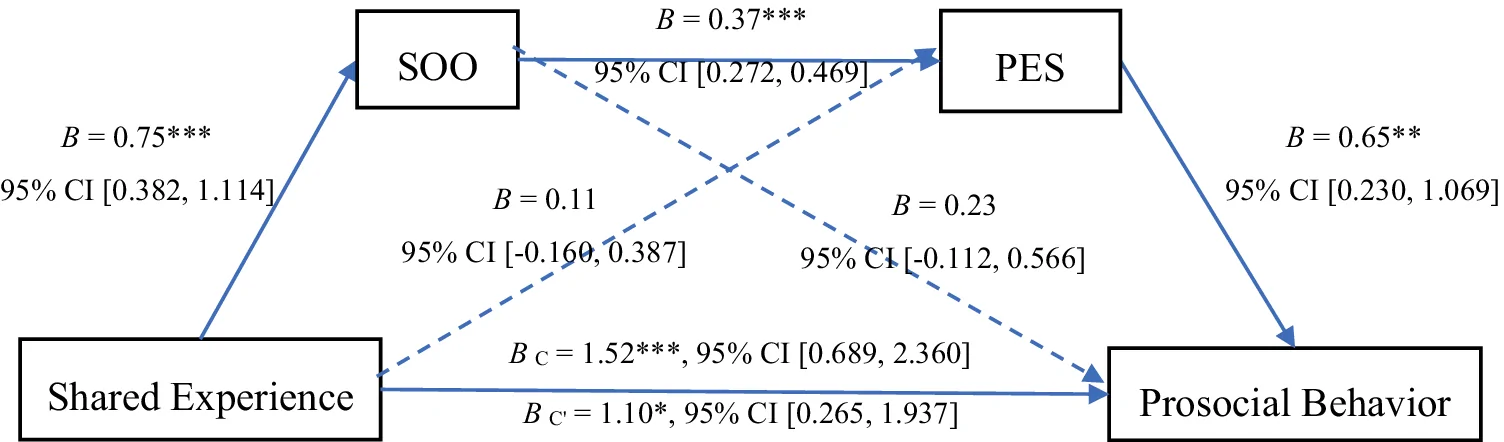
The serial mediation model. *p < 0.05, ^**^p < 0.01, ^***^p < 0.001. Effect sizes are unstandardized coefficients.

**Table 5:** Indirect effects for the hypothesized and reverse serial mediation models.

Indirect effects	Effect (SE)	95% CI
Hypothesized serial model		
Shared experience → SOO → PES → prosocial behavior		
Total indirect effect	0.424 (0.17)	[0.113, 0.779]
Shared experience → SOO → Prosocial behavior	0.169 (0.14)	[−0.092, 0.446]
Shared experience → PES → Prosocial behavior	0.074 (0.09)	[−0.101, 0.273]
Shared experience → SOO → PES → Prosocial behavior	0.180 (0.08)	[0.046, 0.357]
Reverse model		
Shared experience → PES → SOO → Prosocial behavior		
Total indirect effect	0.424 (0.17)	[0.109, 0.783]
Shared experience → PES → Prosocial behavior	0.254 (0.13)	[0.038, 0.551]
Shared experience → SOO → Prosocial behavior	0.119 (0.10)	[−0.060, 0.328]
Shared experience → PES → SOO → Prosocial behavior	0.050 (0.05)	[−0.023, 0.164]

Effect sizes are unstandardized coefficients.

To examine the proposed ordering further, an alternative model with reversed mediator order (shared experience → PES → SOO → prosocial behavior) was examined. The serial indirect effect in the reversed model was not significant. However, the indirect effects through PES alone remained significant, whereas the indirect effect through SOO alone was not significant (see Table 5). These findings provided greater support for the proposed SOO → PES serial pathway than for the reverse serial pathway.

## Discussion

### Study 3 extended the previous findings

The results were also consistent with the hypothesized serial pathway: shared experience predicted greater SOO, which in turn predicted higher PES, and this sequence was associated with increased prosocial behavior. The direct effect of shared experience remained significant, indicating that these variables account for part, but not all, of the observed effect. This pattern aligns with prior research showing that emotional synchrony is associated with increased cooperation and social bonding (Páez et al., 2015; Chung et al., 2024).

The pattern of indirect effects further clarifies this process. While the serial pathway was significant, the indirect effect through PES alone and SOO alone were not significant. In addition, the serial indirect effect in the alternative model with reversed mediator order was not significant. Together, these findings are consistent with the idea that SOO may function as an upstream relational-cognitive condition, whereas PES represents a more proximal socioemotional process linked to prosocial responding.

Methodologically, Study 3 strengthens the present research by experimentally manipulating shared experience and assessing prosocial responding via the Public Goods Game. At the same time, because the mediators were measured in close temporal proximity, stronger conclusions regarding their causal ordering remain premature; the present findings are therefore more consistent with the proposed ordering than conclusive about it. Future research may further examine additional pathways and boundary conditions.

## General discussion

The present research examined how shared emotional experiences shape prosocial responding across both positive and negative contexts, using controlled laboratory paradigms as well as real-world collective events. Across four studies, the findings converged on two primary conclusions. First, experiencing an emotional event together was associated with greater prosocial responding than experiencing individually, and this pattern emerged across different emotional valences. Second, the prosocial consequences of shared experience appeared to operate, at least in part, through a structured psychological pathway in which changes in self–other representation were associated with subsequent emotional alignment with others. Specifically, SOO was associated with higher PES, which in turn predicted greater prosocial responding.

These findings contribute to the literature by highlighting the importance of sharedness as a social property of emotional experience. Traditional approaches to emotion and prosociality often emphasize emotional valence, suggesting that positive emotions broaden cognition and promote helping, whereas negative emotions may sometimes constrain self-regulation or increase aggression (Fredrickson, 1998; Fredrickson et al., 2003; Verona et al., 2002; Dorfman et al., 2014; Twenge et al., 2007; Denson et al., 2011). The present findings suggest that the social consequences of emotional events may depend not only on valence, but also on whether the event is experienced together. Even negatively valenced shared experiences—such as collective mourning or natural disaster exposure—were associated with greater prosociality. This pattern is consistent with research on altruism born of suffering and common fate, which suggests that adversity can strengthen solidarity and motivate helping when experienced in shared social contexts (Staub and Vollhardt, 2008; Whitehouse and Lanman, 2014; Miao et al., 2021).

The present research also refines existing accounts of emotional synchrony. Prior work has consistently shown that synchrony and shared affect are linked to social bonding, cohesion, and cooperation (Wiltermuth and Heath, 2009; Páez et al., 2015; Mogan et al., 2017; Chung et al., 2024). The current findings extend this literature by suggesting that emotional synchrony may not emerge in a psychological vacuum. Rather, it may depend partly on a prior relational-cognitive shift in how others are represented. When individuals perceive reduced boundaries between self and others, others may become more socially relevant and more likely to be treated as targets of concern. In this state, emotional convergence with others may become more likely, and such synchrony may subsequently facilitate cooperative or prosocial action. This interpretation is consistent with the Inclusion of Other in the Self framework and related work showing that perceived self–other closeness promotes helping and identification (Aron et al., 1992; Cialdini et al., 1997).

More broadly, the proposed pathway is compatible with emotional contagion and perception–action accounts, which suggest that reduced self–other boundaries increase susceptibility to others’ emotional states (Hatfield et al., 1993, 1994; Niedenthal, 2007). Within this framework, shared experience may not directly produce prosociality; instead, it may first reshape self–other representation, thereby enabling emotional alignment and social responding. The present findings therefore help clarify how transient shared experiences among unfamiliar individuals can produce meaningful social consequences even in the absence of long-term interaction or strong pre-existing group identity (Wolf et al., 2016; Páez et al., 2015).

Importantly, the observed effects were generally modest in magnitude, but this should not be interpreted as trivial. Prosocial responding in naturalistic settings is multiply determined by situational constraints, available resources, social norms, efficacy beliefs, and individual differences (Batson and Powell, 2003; Piff et al., 2010; Van Doesum et al., 2017). Against this background, even modest unique effects of shared experience or PES may be theoretically meaningful. As noted in classic discussions of effect sizes, small statistical effects can produce substantial real-world consequences when accumulated across repeated social interactions and large populations (Abelson, 1985). This may be especially true during public events, crises, or communal rituals.

The domain-specific pattern across prosocial outcomes is also informative. In Study 2, the strength of the association between PES and prosocial responding varied across outcomes. Different forms of prosocial responding may involve distinct motivational demands, behavioral costs, and situational opportunities, particularly in an ongoing crisis. Thus, the present findings should not be interpreted as indicating an equivalent role of PES across all forms of prosocial behavior; rather, its relevance may vary across behavioral domains.

The serial mediation findings further clarify the psychological process. Although SOO alone did not consistently account for behavioral outcomes, the observed pattern was more consistent with the proposed SOO → PES pathway than with the reverse ordering. One interpretation is that feeling psychologically closer to others may increase individuals’ willingness to cooperate, whereas PES may be more directly associated with prosocial responding. However, because SOO and PES were measured in close temporal proximity, this interpretation should not be taken as establishing their causal or temporal ordering.

Although the present findings should not be taken as direct intervention evidence, they suggest that in contexts marked by social fragmentation, loneliness, or weak interpersonal ties, creating opportunities for shared experience may offer a relatively low-cost route to fostering connection and cooperative motivation. Public rituals, collective commemorations, synchronized activities, and shared media experiences may help individuals feel less isolated and more attuned to others. This possibility is consistent with evidence linking social isolation to poorer psychological and social functioning (Cacioppo and Hawkley, 2009; Holt-Lunstad et al., 2015). In crisis settings, interventions that highlight common experience and shared fate may strengthen willingness to help, volunteer, or coordinate with others.

Several limitations should be acknowledged. Although multiple methods were used, some measures relied on self-report and retrospective recall, which may be influenced by memory bias or social desirability. In addition, the manipulation in Studies 1a and 3 operationalized shared experience as a natural co-experiencing context involving direct co-experience, simultaneous attention, and incidental access to others’ reactions. The present design was not intended to disentangle the unique contribution of each component, which remains an important direction for future research. Moreover, although Study 3 experimentally manipulated shared experience, the mediators were measured in close temporal proximity; therefore, stronger causal claims regarding the precise ordering of SOO and PES should be made cautiously. At this stage, the evidence should be viewed as suggestive rather than definitive.

The scope and generalizability of the present findings also remain limited. The studies focused primarily on transient shared experiences among unfamiliar individuals. The same pathway may operate differently in intimate relationships, long-standing groups, or highly polarized intergroup contexts. In addition, the remaining direct effects suggest that additional mechanisms—such as shared identity, perceived similarity, or collective meaning-making—may also contribute to the social consequences of shared experience. Finally, most of the present studies relied heavily on female university student samples, which limits the generalizability of the findings to broader populations or different demographic groups. Future studies should employ more representative samples to examine whether these cognitive-affective processes operate consistently across diverse social backgrounds.

Future research may build on these findings by examining when shared experience most strongly promotes prosociality, for whom it is most effective, and how long such effects endure. It would also be valuable to compare naturally occurring shared experiences with intentionally designed collective interventions, as well as to test whether digital co-experience can produce similar benefits. More broadly, the present research suggests that moments of shared experience may play an underappreciated role in promoting social connection and prosocial responsiveness in everyday and collective contexts.

## Conclusion

Across laboratory and real-world contexts, shared emotional experiences are associated with greater prosocial responding in both positive and negative settings. The findings are consistent with a cognitive-affective account in which greater self-other overlap may provide an upstream relational-cognitive condition associated with higher perceived emotional synchrony, which in turn is linked to prosocial responding. Although the present studies do not establish a definitive causal or temporal ordering between self-other overlap and perceived emotional synchrony, the overall pattern is consistent with the proposed sequence. More broadly, these findings highlight sharedness as an important social feature of emotional experience and suggest that even relatively incidental, low-interaction co-experiences may influence how individuals relate to and respond to others.

## Data Availability

The raw data supporting the conclusions of this article will be made available by the authors, without undue reservation.
