# Supplementary material for: Shared experiences and prosocial responding: the roles of self-other overlap and perceived emotional synchrony
Source: Front Psychol. 2026 Oct 7;17:1763805. doi: 10.3389/fpsyg.2026.1763805 (PMC13683166; doi:10.3389/fpsyg.2026.1763805)
Supplement: Supplementary file 1 [file Table_1.DOCX]

**Supplementary Materials**

**Study 1a**

**Tables S1–S4, together with Supplementary Note S1, present supplementary analyses for Study 1a.**

Table S1

*Results of Manipulation Checks*

|  |  | *M* | *SD* | *t* |
| --- | --- | --- | --- | --- |
| Pleasure | Shared Experience | 4.56 | 1.01 | 0.78 |
|  | Individual Experience | 4.41 | 1.12 |  |
| Physiological  arousal | Shared Experience | 3.10 | 1.42 | 1.33 |
|  | Individual Experience | 2.74 | 1.59 |  |

**Note. Experience Mode:0=Individual Experience，1=Shared Experience**

Table S2

*Analysis of Control Variables*

|  |  | *M* | *SD* | *t* |
| --- | --- | --- | --- | --- |
| Familiarity | Shared Experience | 2.11 | 1.59 | 0.96 |
|  | Individual Experience | 1.83 | 1.69 |  |
| Preference | Shared Experience | 3.84 | 1.239 | -0.10 |
|  | Individual Experience | 3.86 | 1.169 |  |

**Note. Experience Mode:0=Individual Experience，1=Shared Experience**

Table S3

*Group Differences in Interpersonal Relationship Quality*

|  |  | *M* | *SD* | *t* |
| --- | --- | --- | --- | --- |
| Connectedness | Shared Experience | 3.3 | 1.49 | 1.46 |
|  | Individual Experience | 2.94 | 1.32 |  |
| Trust | Shared Experience | 3.63 | 1.47 | 1.62 |
|  | Individual Experience | 3.21 | 1.48 |  |
| Similarity | Shared Experience | 3.38 | 1.40 | 3.21^*^ |
|  | Individual Experience | 2.61 | 1.35 |  |

**Note.*^*^p* < 0.05, ^**^*p* < 0.01; Experience Mode: 0=Individual Experience，1=Shared Experience**

Table S4

*Exploratory Robustness Analysis Using a Gender-Balanced Subsample in Study 1a*

| *Variable* | *t* | *p* |
| --- | --- | --- |
| Pleasure | 0.32 | .75 |
| Arousal | -0.64 | .58 |
| Familiarity | 0.71 | .48 |
| Preference | 0.31 | .76 |
| Perceived similarity | 2.38* | < .05 |
| Connectedness | 1.056 | .30 |
| Trust | 1.53 | .13 |
| Round 1 choice | 2.41* | < .05 |
| Round 1 earnings | 1.68 | .10 |
| Round 2 earnings | 2.68* | < .05 |
| Total earnings | 1.84 | .07 |

**Note.** **To address the gender imbalance in the full sample, an exploratory robustness analysis was conducted using a gender-balanced subsample. Data from 13 female participants were randomly selected from each condition and combined with data from the 25 male participants, yielding a final subsample of 51 participants. Higher values on the Weak-link Coordination Game indicate stronger cooperative tendencies. p < .05, p < .01.**

**Supplementary Note S1. Robustness Check for Gender Imbalance in Study 1a**

To control for potential gender-related biases stemming from the significant gender imbalance in Study 1, a simple random sampling technique was utilized. Data from 13 female participants selected from both the shared and individual conditions were merged with the data of the 25 male participants to conduct independent samples t-tests on a subsample of 51 individuals. The analysis yielded the following findings (see Table S4). There were no significant differences in induced pleasantness between the shared and individual experience groups, *t*(49) = 0.32, *p* = .75. No significant differences were observed regarding physiological arousal, *t*(49) = -0.64, *p* = .58. Participants in both groups reported comparable levels of familiarity with the music, *t*(49) = 0.71, *p* = .48. Similarly, no significant difference was found regarding preference for the music between the conditions, *t*(49) = 0.31, *p* = .76.

Shared experiencers reported significantly higher perceived similarity with group members compared to those in the individual condition, *t*(49) = 2.38, *p* < .05. No significant differences were detected for other prosocial attitudes, such as connectedness, *t*(49) = 1.06, *p* = .30 or trust , *t*(49) = 1.53, *p* = .13. Significant differences were observed between the conditions regarding the coordination values chosen in the first and second rounds of the game. These round-specific differences were statistically significant, *t*(49) = 2.41, *p* = < 0.05 and *t*(49) = 3.51, *p* < 0.01.The difference in earnings in the first round was marginally significant, *t*(49) = 1.68, *p* = .10. Earnings in the second round differed significantly between the shared and individual conditions, *t*(49) = 2.68, *p* < .05.Total cumulative earnings showed a marginal significance between the conditions, *t*(49) = 1.84, *p* = .07.

These results align with the primary findings of Experiment 1a, even though total earnings did not reach a level of full statistical significance. Critically, the choices in the first and second rounds—where larger numbers reflect a stronger prosocial orientation—differed significantly between conditions. Furthermore, observed differences in earnings persisted across groups.

**Study 1b**

**Tables S5–S6, together with Supplementary Note S2, present supplementary analyses for Study 1b**

Table S5

*Means, Standard Deviations, and Correlations Among Study Variables*

|  |  | M | SD | 1 | 2 | 3 | 4 | 5 | 6 | 7 |
| --- | --- | --- | --- | --- | --- | --- | --- | --- | --- | --- |
| 1 | Gender | 0.63 | 0.49 | 1 |  |  |  |  |  |  |
| 2 | Age | 22.45 | 4.71 | 0.05 | 1 |  |  |  |  |  |
| 3 | Experience mode | 0.60 | 0.49 | -.027** | 0.11 | 1 |  |  |  |  |
| 4 | Negative affect | 2.25 | 0.78 | -0.03 | -0.06 | 0.06 | 1 |  |  |  |
| 5 | Social desirability | 5.97 | 2.13 | -0.06 | 0.14 | 0.22** | -0.13 | 1 |  |  |
| 6 | Self-Other Overlap | 4.87 | 1.59 | -0.08 | 0.02 | 0.15* | 0.07 | 0.01 | 1 |  |
| 7 | Prosocial behavior | 1.64 | 1.37 | -0.02 | 0.17* | 0.25** | 0.09 | 0.15* | 0.06 | 1 |

**Note.**p* < .05, ***p* < .01; gender：0=male，1=female; Experience Mode: 0= Individual Experience，1= Shared Experience.**

Table S6

*Comparison of Self-Other Overlap by Experience Mode*

|  |  | M | SD | t |
| --- | --- | --- | --- | --- |
| Self -Other Overlap | Individual Experience | 4.58 | 1.39 | -1.98^*^ |
|  | Shared Experience | 5.07 | 1.69 |  |

**Note.Experience Mode:0=Individual Experience，1=Shared Experience**

**Supplementary Note S2. Common Method Bias Test in Study 1b**

To assess potential common method bias, Harman’s single-factor test was conducted using all measured variables in an exploratory factor analysis. Two factors with eigenvalues greater than 1.00 emerged, and the first factor accounted for 31.15% of the total variance. This proportion was below the commonly used threshold of 40%, suggesting that common method bias was unlikely to pose a serious concern in Study 1b.

**Study 2**

**Tables S7 and S8, together with Supplementary Note S3, present supplementary analyses for Study 2.**

Table S7

*Hierarchical Regression Analysis of Perceived Emotional Synchrony (PES) on Prosocial Behavior in study2*

|  | Prosocial Behavior | | |
| --- | --- | --- | --- |
|  |  | Model 2 | |
| Variables | Model 1B | *B* | 95%CI |
| Constant | 0.16 | -0.52 | [-1.824,0.62] |
| Age | 0.02 | 0.02 | [-0.002,0.035] |
| Gender | -0.23 | -0.22 | [-0.457,0.021] |
| Empathy | 0.048 | -0.06 | [-0.32,0.231] |
| Need for connection | 0.67^**^ | 0.59^**^ | [0.383,0.781] |
| PES |  | 0.33^**^ | [0.041,0.74] |
| *R*^2^ | 0.25 | 0.27 | |
| *F* | 31.42^**^ | 27.42^**^ | |
| Δ*R*^2^ |  | 0.02 | |
| Δ*F* |  | 8.69 | |

**Note.(1)**p* < .05, ***p* < .01. (2) gender:1 = female; 0 = male**

Table S8

*Hierarchical Regression Analysis of Perceived Emotional Synchrony (PES) on Prosocial Intention in study 2*

|  | Prosocial Intention | | |
| --- | --- | --- | --- |
|  |  | Model 2 | |
| Variables | Model 1*B* | *B* | 95%CI |
| Constant | 0.30 | -0.25 | [-1.266,0.843] |
| Age | 0.01 | 0.01 | [-0.005,0.025] |
| Gender | 0.04 | 0.05 | [-0.161,0.266] |
| Empathy | 0.16 | 0.07 | [-0.146,0.246] |
| Need for Connection | 0.26^**^ | 0.20^*^ | [0.03,0.349] |
| PES |  | 0.26^*^ | [0.088,0.531] |
| *R*^2^ | 0.10 | 0.12 | |
| *F* | 10.06^**^ | 9.73^**^ | |
| Δ*R*^2^ |  | 0.02 | |
| Δ*F* |  | 7.66 | |

**Note.(1)*p < .05, **p < .01.(2) gender:1 = female; 0 = male**

**Supplementary Note S3. Common Method Bias Test in Study 2**

Because all variables were assessed via self-report measures, Harman’s single-factor test was conducted to examine potential common method bias. The exploratory factor analysis revealed two factors with eigenvalues greater than 1.00, and the first factor explained 31.81% of the total variance. This value was below the conventional threshold of 40%, indicating that common method bias was unlikely to substantially affect the present findings.

**Study 3**

**Tables S9- S11 , together with Supplementary Note S4, present supplementary analyses for Study3.**

Table S10

*Means, Standard Deviations, and Correlations Among Study Variables in study 3*

| Variable | M | SD | shared experience | prosocial behavior | PES | SOO |
| --- | --- | --- | --- | --- | --- | --- |
| 1. Shared experience | 0.51 | 0.5 | 1 |  |  |  |
| 2. Prosocial behavior | 5.26 | 3.15 | .24** | 1 |  |  |
| 3. PES | 3.57 | 1.1 | .18** | .31** | 1 |  |
| 4. SOO | 2.62 | 1.39 | .27** | .26** | .48** | 1 |

**Note.** **Experience condition was coded as 0=Individual Experience,1=Shared Experience.***p < .001, **p < .001, *p < .05.**

Table S11

*Direct and Total Effects of Shared Experience on Prosocial Behavior in study 3*

| Relationship | Coefficient | SE | *t* | *p* | 95% CI [LL, UL] |
| --- | --- | --- | --- | --- | --- |
| Total Effect (c) | 1.52 | 0.42 | 3.60 | 0 | [0.6888, 2.3592] |
| Direct Effect (c′) | 1.10 | 0.42 | 2.60 | .01 | [0.2646, 1.9362] |

**Note. All values are unstandardized coefficients.**

**Supplementary Note S4. Pilot study of study 3**

**Participants**

37 Chinese undergraduate and graduate students (10 males, 27 females) recruited from Tsinghua University and nearby universities in Beijing via online advertisement participated in the experiment as paid volunteers. The mean age of participants was 23.19 years (*SD*=3.081).

All of them are healthy with normal hearing, normal or corrected-to-normal vision. Informed consent was obtained from all participants prior to the study according to procedures approved by the local Ethics Committee of Tsinghua University.

**Stimuli**

We used 6 video clips to elicit intended emotional states. All clips were selected via interviews and evaluations. We interviewed ten filmmakers who work in CCTV movie Channel and have seen more than 3000 movies, to recommend film clips that make them feel sad. Then we showed the top 6 highest rated sad clips from the interviews to 37 participants. The sad clips are cut from film*s The Champs*, *A.I*., *Departures*, *Shoplifters*, *Go Away Mr.Tumour*, and *Quill*. Their lengths range from 60 to 300 seconds.

For all the film clips intended to elicit sad emotions, a full scene was included so the participants could have a better understanding and become more engaged with the material. For the film clips containing non-Chinese dialogs, Chinese subtitles were added to guarantee a full understanding of the contents.

**Emotion Measures**

After viewing each video clip, participants reported their current emotional state on arousal, sad emotions with the materials. Responses were made on a 5-point rating scale ranging from *not at all* to *extremely strong*.

**Procedure**

The experiments were carried out in the laboratory environment with controlled illumination (at a normal office illumination level). The stimuli were displayed on an LCD monitor (22- inch, DELL, USA) with a 60 Hz refreshing rate. Upon arrival, participants completed informed consent and were told that the study examines the movie viewing experience.

Before the clip presentation, participants were instructed to immerse themselves in the scenario and feeling depicted in the clips. After viewing each video clip, participants rated their current emotional state with 5-point Likert scale on 3 items measuring their arousal level, degree of sad emotions with the material. After each rating process, they will have a one-minute break, when they were asked to try their best to calm down and forget the previous clip, and then when ready, press the space button to watch the next clip. The movie orders were counterbalanced.

After viewing all 6 sad clips, participants were instructed to sort the clips based on the intensity of sad emotion.

**Results**

Repeated Measurement ANOVA was conducted to check whether each type of video clips actually elicit the intended emotions.

**Arousal**

When comparing the arousal of clips, results showed a significant difference among different clips, *F* (5, 180) =16.292, *p*<0.01, *η*^2^=0.312.

**Intensity of emotions**

Results showed a significant difference among different clips, *F* (5, 180) =16.292, *p*<0.01, *η*^2^=0.312.

**Rank**

We endow different weight to the rank of movie clips with the first weighting 5, the second one weighting 3, the third weighting 1, and the rest weighting 0. Then we calculated the average rank of each clip. The mean rank was shown in Table S12 .

Table S12

*means of arousal, emotional intensity and rank of clips*

|  | Arousal | Happiness | Sadness | Rank |
| --- | --- | --- | --- | --- |
| Go Away Mr.Tumour | 3.784 | 1.838 | 3.919 | 2.46 |
| Departures | 3.405 | 1.649 | 3.919 | 1.89 |
| The Champs | 3.189 | 1.838 | 3.622 | 1.62 |
| A.I. | 3.216 | 2.189 | 3.595 | 1.49 |
| Quill | 2.216 | 2.216 | 3.216 | 0.62 |
| Shoplifters | 2.595 | 2.027 | 3.189 | 0.92 |

**Conclusion**

The clips from Go Away Mr.Tumour, Departures, and The Champs were selected.
